# Towards Biohybrid Lung Development: Establishment of a Porcine In Vitro Model

**DOI:** 10.3390/membranes12070687

**Published:** 2022-07-03

**Authors:** Simon Schlör, Michael Pflaum, Klaus Höffler, Christian Kühn, Axel Haverich, Bettina Wiegmann

**Affiliations:** 1 Department for Cardiothoracic, Transplantation and Vascular Surgery, Hannover Medical School, Carl-Neuberg-Str. 1, 30625 Hannover, Germany; schloer.simon@mh-hannover.de (S.S.); pflaum.michael@mh-hannover.de (M.P.); hoeffler.klaus@mh-hannover.de (K.H.); kuehn.christian@mh-hannover.de (C.K.); haverich.axel@mh-hannover.de (A.H.); 2 Lower Saxony Center for Biomedical Engineering, Implant Research and Development (NIFE), Stadtfelddamm 34, 30625 Hannover, Germany; 3 German Center for Lung Research (DZL), Carl-Neuberg-Str. 1, 30625 Hannover, Germany

**Keywords:** biohybrid lung, hollow fiber membrane, endothelialization, hemocompatibility, porcine endothelial cells

## Abstract

Lung transplantation (LTx) is the only curative therapy option for patients with end-stage lung diseases, though only available for chosen patients. To provide an alternative treatment option to LTx, we aim for the development of an implantable biohybrid lung (BHL) based on hollow fiber membrane (HFM) technology used in extracorporeal membrane oxygenators. Crucial for long-lasting BHL durability is complete hemocompatibility of all blood contacting surfaces, which can be achieved by their endothelialization. In continuation to successful in vitro investigations using human endothelial cells (ECs), indicating general feasibility, the appropriate porcine in vivo model needs to be prepared and established to fill the translational data gap prior to patient’s application. Therefore, isolation of porcine ECs from carotid arteries (pCECs) was established. Following, pCECs were used for HFM endothelialization and examined under static and dynamic conditions using cell medium or heparinized blood, to assess their proliferation capacity, flow resistance and activation state, especially under clinically relevant conditions. Additionally, comparative hemocompatibility tests between native and endothelialized HFMs were performed. Overall, pure pCECs formed a viable and confluent monolayer, which resisted applied flow conditions, in particular due to physiological extracellular matrix synthesis. Additionally, pCECs remained the non-inflammatory and anti-thrombogenic status, significantly improving the hemocompatibility of endothelialized HFMs. Finally, as relevant for reliable porcine to human translation, pCECs behaved in the same way as human ECs. Concluding, generated in vitro data justify further steps towards pre-clinical BHL examination, in particular BHL application to porcine lung injury models, reflecting the clinical scenario with end-stage lung-diseased patients.

## 1. Introduction

Even before the COVID-19 pandemic, lung diseases were one of the greatest threats to global health. In 2019, chronic obstructive pulmonary diseases accounted for approximately 6% of global deaths, making them the third leading cause of death worldwide [1]. In the field of terminal lung diseases, symptomatic therapy has improved through the years, but nevertheless, the only curative therapy option remains the lung transplantation (LTx), which is still a critical surgical procedure fraught with complications. As a result, many patients in urgent need for a LTx are not even considered, for example due to certain co-morbidities or advanced age [2,3]. Additionally, despite the contemplated strict indication limitations to transplant only patients with low or moderate risks [4], a high complication rate is evident, leading to a devastating five-year survival of 59% [5]. Moreover, a permanent shortage of donor organs further exacerbates the situation [6].

Another major problem for patients with end-stage lung diseases is the unavailability of a reliable long-term lung support or rather replacement system as alternative therapy option to LTx, such as dialysis for end-stage renal failure [7] or a left ventricular assist device for severe heart failure [8,9]. In the event of respiratory insufficiency, artificial ventilation is initiated, but if insufficient, it needs to be expanded by the extracorporeal membrane oxygenation (ECMO). ECMO technology is based on poly-4-methyl-1-pentene (PMP) hollow fiber membranes (HFMs), in which the gas flow is applied and on the outside the blood passes by for gas exchange [10]. However, due to this unavoidable contact between the patient’s blood and the artificial PMP-HFM surface, coagulation and inflammation activation occurs, resulting in a time-limited lung support for a few weeks. As a consequence, ECMO therapy requires a strict anticoagulation regime, which is highly prone to complications such as thromboembolisms and partly lethal hemorrhage [11], needing intensive medical care. Accordingly, ECMO therapy will only be applied as a last resort, if a bridge to successful transplantation or a bridge to recovery is secured [12]. 

Overall, these facts underline the compelling necessity for the development of a biocompatible long-term lung assist device for both, as alternative and bridge to LTx. The biohybrid lung (BHL), which we aim for can fulfill these required conditions. In this context, the already clinically established ECMO principle represents the technology of choice. Nevertheless, for long-term use, it is crucial to improve the hemocompatibility of the HFMs used in contemporary ECMO. Therefore, endothelial cell (EC) colonization of the HFMs seems to be an ideal method for hemocompatibility improvement, since the endothelium not only provides a barrier between the blood and the artificial surface, but also actively expresses anti-thrombogenic surface molecules [13]. 

The general feasibility of the BHL has already been demonstrated, as successful endothelialization of PMP-HFMs [14,15,16,17] significantly improved their hemocompatibility, in particular indicating significantly lower platelet aggregation compared to the native, clinically used PMP-HFMs [18]. Hereinafter, the viable, flow-resistant, anti-inflammatory and anti-thrombogenic endothelial monolayer could be maintained for several weeks [19] without any significant gas exchange impairment [20]. Focusing on appropriate cell sources, induced pluripotent stem cell-derived ECs [21] and MHC-silenced ECs were used for PMP-HFM endothelialization, preventing allogenic cell rejection [22]. Finally, for clinically highly relevant “off-the-shelf” use of the BHL, hypothermic storability was shown [23]. 

In order to fill the translational gap, various aspects of the BHL need to be thoroughly tested in large animal studies prior to the patient’s application. Therefore, the appropriate porcine model needs to be prepared. Thus, applying the BHL in the porcine model entails that the biological components, i.e., the ECs, need to come from porcine sources, and not as in all our preceding studies from human sources, in order to prevent potential rejection processes to the foreign, xenologous material. Furthermore, it needs to be elucidated if porcine ECs respond to the BHL environment in the same manner as human ECs, in order to allow the transfer of reliable information to be implemented in the development of the human BHL prototype. Accordingly, we identified an isolation protocol for highly pure porcine endothelial cells to be used for PMP-HFM endothelialization for subsequent qualitative, quantitative and functional analysis, including flow resistance and hemocompatibility tests using porcine blood. 

## 2. Materials and Methods

### 2.1. Porcine Endothelial Cell Isolation and Cultivation

#### 2.1.1. Cell Isolation

Porcine carotid arteries were aseptically excised from pigs directly after sacrifice in accordance with the animal welfare act. During transport to the laboratory, blood vessels were stored in 100 mL Tiprotec per blood vessel (Dr. F. Köhler Chemie, Bensheim, Germany) at 4 °C.

For endothelial cell (EC) isolation, an enzymatic detachment protocol was utilized. For this purpose, a three-way stopcock (B. Braun, Melsungen, Germany) was attached to one end of the vessel using cable ties. Then, the blood vessel was rinsed with 10 mL Gibco phosphate buffered saline (PBS, Thermo Fisher Scientific, Waltham, MA, USA) to remove any remaining blood components. After closing the opposing end of the vessel with an umbilical clamp (Dahlhausen, Köln, Germany), 1 to 2 mL of a 1% collagenase solution (Type CLS, 240 U/mg, Biochrom, Berlin, Germany) was filled into the artery via the three-way stopcock until the vessel was visibly inflated. Following enzymatic digestion for 20 min at room temperature (RT), the three-way stopcock was opened over a 50 mL polypropylene (PP) tube and the emerging enzyme-cell solution was placed directly into the tube. In order to detach further cells, the blood vessel was then cut open a few millimeters above the umbilical clamp and was flushed with 20 mL Gibco Dulbecco’s Modified Eagle Medium (DMEM, Thermo Fisher Scientific, Waltham, MA, USA) containing 10% fetal bovine serum (FBS, Capricorn Scientific, Ebsdorfergrund, Germany), which was also collected in the PP-tube. The obtained porcine carotid artery endothelial cells (pCECs) in suspension were sedimented by centrifugation at 300× *g* for five minutes and resuspended in endothelial cell growth medium (EGM2, Lonza, Basel, Switzerland).

#### 2.1.2. Cell Cultivation on Tissue Culture Plastic (TCP)

pCECs were seeded in TCP flasks (Delta surface, Nunc, Rochester, NY, USA) and incubated under standard culture conditions (37 °C, 21% O_2_ and 5% CO_2_ with saturated humidity). Growth medium EGM2 was replaced every 48 h and pCEC populations were detached upon reaching 80% confluence using Trypsin/EDTA (0.05%/0.02% (*w*/*v*), Biochrom, Berlin, Germany). ECs were passaged repeatedly and reseeded each time at a density of 8 × 10³ cells/cm². Cell numbers were determined using a CASY TT cell counting device (OLS, Bremen, Germany) and population doubling times for passage two up to passage nine were calculated using the following equation:

Population doubling time = t × ln (2)/ln (N(t)/N(0))

where t = cultivation time in hours, N(t) = number of cells at time t, and N(0) = number of seeded cells at time 0.

### 2.2. Characterization of the Isolated pCECs

#### 2.2.1. Flow Cytometry Analysis

A MACSQuant Analyzer 10 (Miltenyi Biotec, Bergisch Gladbach, Germany) was used for flow cytometry analysis. pCECs were detached from the TCP using Trypsin/EDTA (0.05%/0.02% (*w*/*v*)) and labeled with anti-rat CD31 Vio-Bright FITC antibodies (Miltenyi Biotec, Bergisch Gladbach, Germany) according to the manufacturer’s instructions. REA Control antibodies, human IgG1 (Miltenyi Biotec, Bergisch Gladbach, Germany), served as isotype control. All data were collected from 2 × 10^4^ events per sample and analyzed using the flow cytometry analysis software Flowlogic for Windows (FlowLogic, Mentone, Australia).

#### 2.2.2. Staining of pCECs on TCP for Fluorescence Microscopy Assessment

For immunofluorescence microscopic analysis of the endothelial specific intercellular junction protein VE-Cadherin and the extracellular matrix constituent Fibronectin, four-well chamber slides (Thermo Fisher Scientific, Waltham, MA, USA) were endothelialized with pCECs. After fixation with 4% paraformaldehyde for 10 min, samples were washed four times with PBS and forwarded into a solution containing 0.25% Triton X-100 in Tris-buffered saline supplemented with 5% donkey serum for 20 min at RT, to permeabilize the ECs and block unspecific binding sites. After washing three times with PBS, samples were incubated sequentially with the primary and the secondary antibodies, for one hour each at RT in the dark with a washing step in between. Antibodies were diluted in PBS containing 1% bovine serum albumin (staining buffer). A complete list of the antibodies used and the respective dilutions is provided in Table 1. After incubation with the secondary antibody, the sections were washed three times with PBS, and for nucleus staining, Hoechst 33342 was diluted with the staining buffer to a concentration of 10 µg/mL and was added to the samples for 15 min at RT in the dark. After washing another three times, all specimens were embedded using the Dako Immunomount mounting medium (Agilent, Santa Clara, CA, USA) and analyzed with the fluorescence microscopes Discovery V.8 (Zeiss, Jena, Germany) and AxioVert A1 (Zeiss, Jena, Germany). Pictures were taken with the AxioCam ICm1 (Zeiss, Jena, Germany). As controls, sections with isotype-matching antibodies were used.

Moreover, monolayer integrity and viability were assessed by staining for 20 min in a 1 µM Calcein-am solution (Thermo Fisher Scientific, Waltham, MA, USA) and subsequent visualization using the fluorescence microscope AxioVert A1.

#### 2.2.3. Gene Expression Analysis via RT-PCR and Quantitative Real-Time qRT-PCR

RNA of lysed pCECs was isolated using the NucleoSpin II Kit (Marchery-Nagel, Düren, Germany) and DNAse (Qiagen, Hilden, Germany) digestion to remove genomic DNA following the manufacturer’s instructions. The RNA concentration was determined spectrophotometrically by employing the nucleic acid quantification device Nanodrop 2000 (Thermo Fisher Scientific, Waltham, MA, USA) and equal amounts of RNA were transcribed into cDNA with the RevertAid First Strand cDNA Synthesis Kit (Thermo Fisher Scientific, Waltham, MA, USA) using random hexamer primer. The obtained cDNA was diluted 1:5 for further analysis.

To confirm the successful cDNA synthesis and the pCEC’s endothelial genotype, RT- PCR was performed using the GoTaq Polymerase Kit (Promega, Madison, WI, USA) and a peqStar Thermocycler (VWR, Rednor, PA, USA). The amplification products were imaged under UV fluorescence after separation in a 1.5% agarose gel with 5% peqGreen (VWR, Rednor, PA, USA). For real-time qRT-PCR reactions, the PowerUP SYBR Green Master Mix (Thermo Fisher Scientific, Waltham, MA, USA) was applied using the Light Cycler 96 (Roche, Basel, Switzerland). All data were normalized to ß-Actin as a housekeeping gene and analyzed using the ΔCt-Method.

To assess the reaction of pCECs towards an inflammatory stimulus, confluent monolayers on TCP were exposed to EGM2 containing 10 ng/mL TNFα (Bachem, Bubendorf, Switzerland) for six hours under standard culture conditions to deliberately induce the pro-inflammatory and pro-thrombogenic EC genotype. Cells incubated in normal EGM2 served as controls. After the incubation period, culture medium was removed and the cells were forwarded to gene expression analysis via real-time qRT-PCR.

The primer pairs used for RT-PCR and real-time qRT-PCR can be found in Table 2.

### 2.3. Endothelialization of Gas Exchange HFMs with pCECs

Heparin and Albumin (H/A) coated and 1.8 × 3.2 cm (40 parallel aligned hollow fibers) sized patches were trimmed from the original HFM of the iLA membrane ventilator (Xenios, Heilbronn, Germany). After sterilization with ethylene oxide, the patches were endothelialized as described previously [21]. In brief, HFM patches were sandwiched between two custom made polycarbonate frames and three patches each were placed into a 50 mL syringe (B. Braun, Melsungen, Germany), which was filled with 23 mL EC suspension (9 × 10^5^ ECs/mL in EGM2). After venting the syringe, it was revolved along the longitudinal axis at one rotation per minute for four hours at 37 °C to allow EC adhesion. After removing the frames from the syringe, they were incubated for 24 h under standard conditions in culture dishes filled with EGM2. The EGM2 was then changed, and the frames were turned over and incubated again for 24 h.

#### 2.3.1. Immunofluorescence Microscopy of pCECs on HFMs

For immunofluorescence microscopic analysis of the endothelialized HFMs, pieces of 0.8 cm diameter were excised using a skin biopsy punch cutter (kai Europe, Solingen, Germany). Subsequently, staining of VE-Cadherin and Fibronectin was performed as described in Section 2.2.2.

#### 2.3.2. Gene Expression Analysis via Real-Time qRT-PCR

In order to comparatively analyze the gene expression of unstimulated and TNFα-stimulated pCECs on HFMs, real-time qRT-PCR was utilized, as described in Section 2.2.3.

### 2.4. Assessment of Endothelialized HFMs Exposed to Flow Conditions Using Culture Medium

Endothelialized HFMs were exposed to flow conditions inside a custom-made flow chamber that was hooked up to a mock circuit, as described previously [14]. Briefly, EGM2 medium was recirculated through the flow chamber and the reservoir (100 mL glass bottle), both placed in the incubator, using a peristaltic pump (ISM 404B, Ismatec, Opfikon, Switzerland). The gas exchange between the medium and the incubator atmosphere (21% O_2_ and 5% CO_2_) was allowed through a 0.22 µm syringe filter (Sartorius, Göttingen, Germany) attached to the reservoir lid. Perfusion was performed with flow rates of 15 mL/min for 24 h or rather 60 mL/min for six hours. For both dynamic conditions, static endothelialized controls were cultivated in parallel for the same time periods.

A schematic overview of the mock circuit and the preceding experiments can be found in the Supplementary Materials (Supplementary Figure S1).

#### 2.4.1. Fluorescence Microscopy of Endothelialized HFMs after Flow

Directly after completion of perfusion, HFMs were dissembled from the flow chambers and monolayer integrity and viability were assessed by Calcein staining as described in Section 2.2.2. This procedure was also used for the static controls.

#### 2.4.2. Gene Expression Analysis via Real-Time qRT-PCR

In case of the 24-h group, both the statically and dynamically cultured samples were subjected to gene expression analysis by real-time qRT-PCR, as described in Section 2.2.3.

### 2.5. Hemocompatibility Testing of Non-Endothelialized versus Endothelialized HFMs

In order to compare the hemocompatibility of non-endothelialized and endothelialized HFMs, the above-described mock circuit was utilized. Instead of cell medium, 100 mL of porcine whole blood, which was anticoagulated with unfractionated heparin (Rotexmedica, Trittau, Germany), was used in each flow chamber circuit to perfuse the HFMs for six hours at a flow rate of 60 mL/min.

#### 2.5.1. Fluorescence Microscopy of Endothelialized HFMs after Blood Flow

In case of the endothelialized HFMs, adherent pCECs were labeled in serum free EGM2 using 25 µM cell tracker red dye (Thermo Fisher Scientific, Waltham, MA, USA) 45 min prior to blood flow application. After flow application, endothelialized HFMs were transferred into EGM2 and examined with the AxioVert A1 fluorescence microscope to assess the monolayer integrity. Endothelialized HFMs cultivated statically during perfusion time and dyed at the same time served as controls.

#### 2.5.2. Thrombus Formation Assessment via Photographs and Scanning Electron Microscopy (SEM)

At the end of perfusion, HFMs were removed from the flow chambers and washed in Gibco Dulbecco’s Balanced Salt Solution (DPBS, Thermo Fisher Scientific, Waltham, MA, USA) to remove non-adherent blood residues. For the macroscopic assessment, HFMs in DPBS were photographed. Using a skin biopsy punch cutter, circular pieces of 0.8 cm diameter were taken from the endothelialized and non-endothelialized HFMs and fixated for 24 h in a SEM fixation buffer containing 150 mM HEPES, 1.5% PFA and 1.5% Glutaraldehyde. After fixation, a graded series with 30%, 50%, 70%, 90% and 100% (*v*/*v*) of EtOH was used for dehydration. For further SEM preparation, samples underwent critical point drying (CPD 030, Balzers, Balzers, Liechtenstein) and were sputtered with gold (Polaron High Resolution Sputter Coater E 5400, Polaron Equipment Ltd., Watford Herts, UK). Imaging was done with the SEM 505 (Philips, Eindhoven, The Netherlands).

#### 2.5.3. Quantification of Blood Parameters Associated with Thrombus Formation

Blood samples were collected from the mock circuits into different blood collection tubes directly before and after six hours blood perfusion of the endothelialized and non-endothelialized HFMs. Blood was collected in citrate tubes (1:10) 2.9 mL (Sarstedt, Nürmbrecht, Germany) for D-Dimer measurement using turbidimetry on the Atellica COAG 360 (Siemens, München, Germany). Determination of the thrombocyte count was carried out with blood collected in K3 EDTA 1.6 mL tubes (Sarstedt, Nürmbrecht, Germany), using impedance measurement on the XN-10 (Sysmex, Kobe, Japan). The relative change in D-Dimer concentrations and thrombocyte counts was expressed in percentages, which were calculated by subtracting the values obtained after flow exposure from the corresponding baseline values before flow divided by the baseline values and multiplied by 100%.

### 2.6. Statistical Analysis

All data are expressed as mean with standard deviation (SD). Comparison between two groups was done using the unpaired, two-tailed *t*-test, whereas statistical significance between multiple groups was checked via one-way ANOVA with correction for multiple comparisons using the Šídák test. All analyses were performed using GraphPad Prism 9 for Windows (GraphPad Software, San Diego, CA, USA).

## 3. Results

### 3.1. High Expansion Capacity and Purity of Endothelial Cells Isolated from Porcine Carotid Artery (pCECs) 

**Figure 1:**
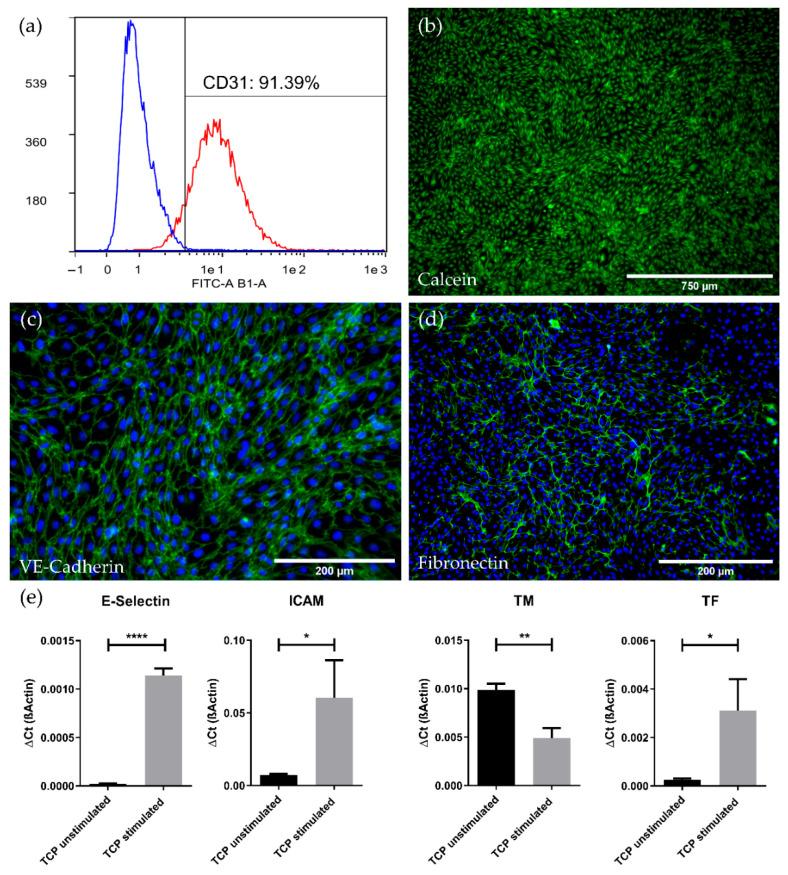
Characterization of isolated pCECs. (**a**) Histogram view of cultured pCECs, analyzed via flow cytometry for the expression of CD31 (red) versus the IgG control (blue); (**b**) Calcein staining (green) of the confluent and viable pCECs monolayer on TCP with a characteristic cobblestone morphology; Immunofluorescence microscopy of pCECs on TCP for the detection of (**c**) the EC-specific junction protein VE-Cadherin (green) and (**d**) the extracellular matrix protein fibronectin (green); (**c**,**d**) Corresponding nuclei were counterstained with Hoechst 33342 (blue); (**e**) Real-time qRT-PCR expression analysis of inflammatory (E-Selectin and ICAM) and thrombogenic state (TM and TF) marker genes with and without TNFα stimulation. Gene expression levels of the stimulated group were compared to its respective unstimulated control group using an unpaired *t*-test. TCP: Tissue Culture Plastic; stimulated: six hours TNFα exposure; unstimulated: control group without TNFα exposure. Results are given as mean with SD (*n* = 3) (* *p* < 0.05; ** *p* < 0.01; **** *p* < 0.0001).

After isolation from porcine carotid arteries, the endothelial cell (EC) population showed a robust proliferative capacity for more than eight passages, with an average population doubling of 41 h. When assessed after seven passages, flowcytometry analysis revealed that more than 91% of the cells were still positive for endothelial specific CD31 (Figure 1a), while staining of adherent cells with Calcein attested viability and the preserved typical endothelial cobblestone morphology (Figure 1b). Representative phase contrast pictures also verified endothelial cell morphology (Supplementary Figure S2b). Furthermore, RT-PCR confirmed the endothelial genotype by detection of the endothelial specific transcripts for CD31, vWF and VE-Cadherin (Supplementary Figure S2a). The confluence of the endothelial monolayer was demonstrated by immunofluorescence microscopic detection of the endothelial-specific intercellular junction protein VE-Cadherin (Figure 1c). In addition, immunofluorescence staining also revealed the deposition of fibronectin on the TCP, indicating physiological de novo production of extracellular matrix by the pCECs (Figure 1d). Real-time qRT-PCR evidenced that ECs resided in a non-activated state, as significant higher expression levels of the inflammatory genes E-Selectin (*p* < 0.0001) and ICAM (*p* < 0.05) and upregulated expression of the pro-thrombogenic gene TF (*p* < 0.05), were found after deliberate EC activation by TNFα stimulation (Figure 1e). Conversely, the gene expression for the anti-thrombogenic TM also showed the anticipated significant level decrease upon TNFα activation (*p* < 0.01).

### 3.2. pCECs Form a Confluent and Non-Activated Neo-Endothelium on HFMs 

#### 3.2.1. pCECs Adherent to HFMs Grow to Confluence and Generate Extracellular Matrix

**Figure 2:**
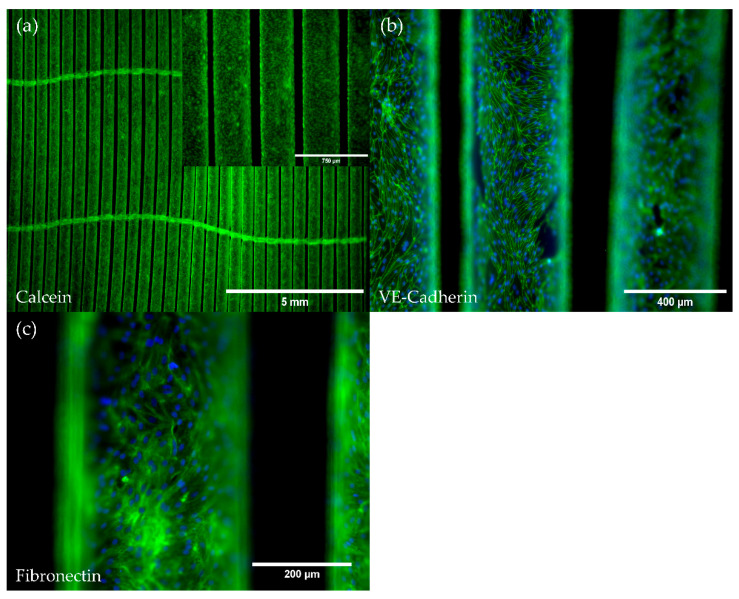
Viability and immunofluorescence staining of pCECs on HFMs. (**a**) Calcein staining of viable pCECs (green) forming a confluent monolayer on the HFM; (**a**) Insert depicts higher magnification; (**b**,**c**) Immunofluorescence staining for the detection of (**b**) VE-Cadherin (green) and (**c**) the de novo synthesized extracellular matrix protein fibronectin (green); (**b**,**c**) Corresponding nuclei were counterstained with Hoechst 33342 (blue).

Upon application of our HFM endothelialization protocol, Calcein staining revealed a viable and confluent endothelial monolayer covering the whole fiber surfaces and showing the endothelial specific cobblestone morphology (Figure 2a). Moreover, comparable to the monolayer on TCP, the intercellular cell junction protein VE-Cadherin, detected via immunofluorescence staining, was ubiquitously expressed throughout the complete monolayer that enveloped all HFM fibers (Figure 2b). Furthermore, immunofluorescence detection also indicated the de novo production of the basement membrane extracellular matrix protein fibronectin (Figure 2c). 

#### 3.2.2. pCECs on HFMs Remain in the Non-Inflammatory and Non-Thrombogenic Genotype

**Figure 3:**
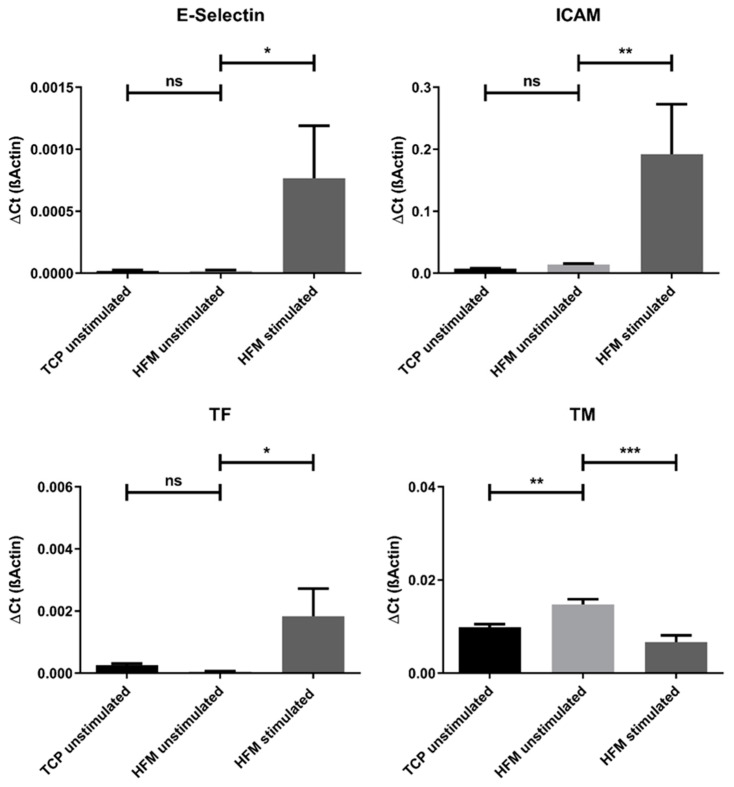
Gene expression analysis for the assessment of the inflammatory and thrombogenic genotype of pCECs on HFMs. Expression levels of the inflammatory (E-Selectin and ICAM) and thrombogenic state (TM and TF) marker genes were compared between untreated pCECs on TCP versus HFMs and pCECs on HFMs stimulated with TNFα. A one-way ANOVA with correction for multiple comparisons (Šídák test) was used to compare these three groups as indicated in the figure. TCP: Tissue Culture Plastic; HFM: Hollow Fiber Membrane; stimulated: six hours of TNFα exposure; unstimulated: control group without TNFα exposure. Results are given as mean with SD (*n* = 3) (* *p* < 0.05; ** *p* < 0.01; *** *p* < 0.001; ns = no significance).

In order to assert that the endothelial cell genotype was preserved in a non-activated state upon seeding and cultivation of the pCECs on the HFM substrate, we performed an expression analysis of the inflammatory marker genes E-Selectin and ICAM, as well as of the thrombogenic state markers TM and TF and compared the results with those of pCECs grown on TCP. No significant differences in the expression levels of the mentioned inflammatory and thrombogenic state marker genes could be detected, except for TM, which was slightly but significantly upregulated in pCECs on the HFM (*p* < 0.01). However, as seen with pCECs seeded on TCP, deliberate TNFα activation still resulted in a significant physiologic regulation of the above-mentioned activation marker genes. (Figure 3).

### 3.3. pCEC Monolayers on HFMs Resist Flow Conditions and Show Typical Physiological Responses on Gene Expression Level

#### 3.3.1. Flow Conditions Do Not Affect Monolayer Integrity

**Figure 4:**
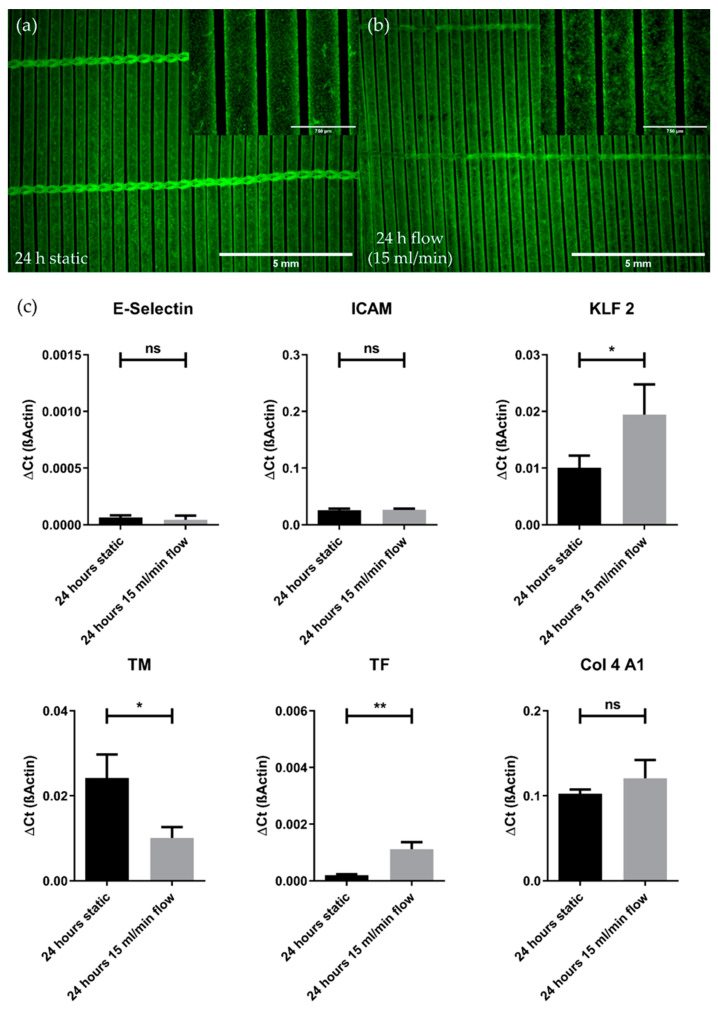
Comparison of pCECs under static and flow conditions. (**a**,**b**) Calcein staining of viable pCECs (green) after 24 h of static (**a**) or dynamic (15 mL/min flow) (**b**) conditions; (**c**) qRT-PCR analysis of flow-exposed pCECs regarding inflammatory activation (E-Selectin and ICAM), shear stress response (KLF 2), thrombogenic state (TM and TF) and matrix synthesis (Col 4 A1). Gene expression levels of the flow-exposed group were compared to the static control groups using an unpaired *t*-test. For abbreviations of gene names, see Table 2. Results are given as mean with SD (*n* = 3) (* *p* < 0.05; ** *p* < 0.01; ns = no significance).

When assessed for the integrity and viability of the endothelial monolayer after flow exposure (15 mL/min) for 24 h using culture medium, fluorescence microscopy images revealed the persistence of a viable and predominantly confluent endothelial monolayer on the HFMs (Figure 4b). In direct comparison with the respective control that was cultivated in parallel under static culture conditions, sporadic and negligible detachment of single pCECs after flow exposure was noticed (Figure 4a). 

#### 3.3.2. Flow Conditions Lead to Physiological Gene Expression Changes While Not Promoting the Pro-Inflammatory Genotype

The impact of flow conditions on pCECs was also examined on gene expression levels (Figure 4c). When compared to static controls, the analyzed genes, indicative for the pro-inflammatory genotype, E-Selectin (*p* = 0.4470) and ICAM (*p* = 0.7166), were not significantly different expressed in pCECs after flow exposure, indicating that flow conditions did not result in the activation of pCECs on HFM. With regard to the anti-thrombogenic genotype, TM was significantly downregulated (*p* = 0.0156) upon 24 h under flow conditions, while TF expression was found to be significantly increased (*p* = 0.0036). Moreover, the capability of pCECs to react physiologically towards shear stress was observed by the significant upregulation of the shear stress marker KLF2 after flow application (*p* = 0.0474). Finally, the expression of COL4A1 as an indicator for extracellular matrix synthesis was not significantly altered after flow application for 24 h (*p* = 0.2327).

### 3.4. The pCEC Monolayer on HFMs Is Capable to Withstand Clinically Relevant Blood Flow Conditions 

**Figure 5:**
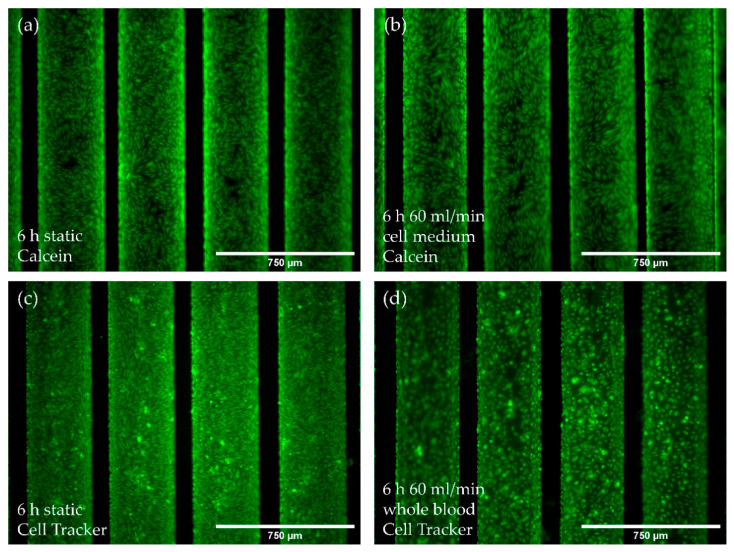
Analysis of EC monolayer integrity after culture medium or blood flow exposure for six hours. (**a**,**b**) Calcein staining (green) of pCECs on HFMs that (**a**) were kept statically or were exposed to (**b**) flow conditions using culture medium (60 mL/min); (**c**,**d**) Cell Tracker staining (green) of pCECs on statically cultivated HFMs (**c**) or on 60 mL/min blood flow exposed HFMs (**d**).

Next, we assessed the pCEC monolayer’s resilience under elevated flow conditions (60 mL/min) using culture medium compared to porcine whole blood for six hours. Comparing the confluence of the endothelialized HFMs receiving flow application with culture medium at 60 mL/min (Figure 5b) to control HFMs that were kept statically in parallel (Figure 5a), no evident cell loss was detectable. The same holds true for flow exposure experiments using whole blood. Here, cell tracker stained pCECs were still evenly distributed all over the HFM, with only few detached cells within the predominantly confluent monolayer (Figure 5d). 

### 3.5. Endothelialization Significantly Improves HFM Hemocompatibility

**Figure 6:**
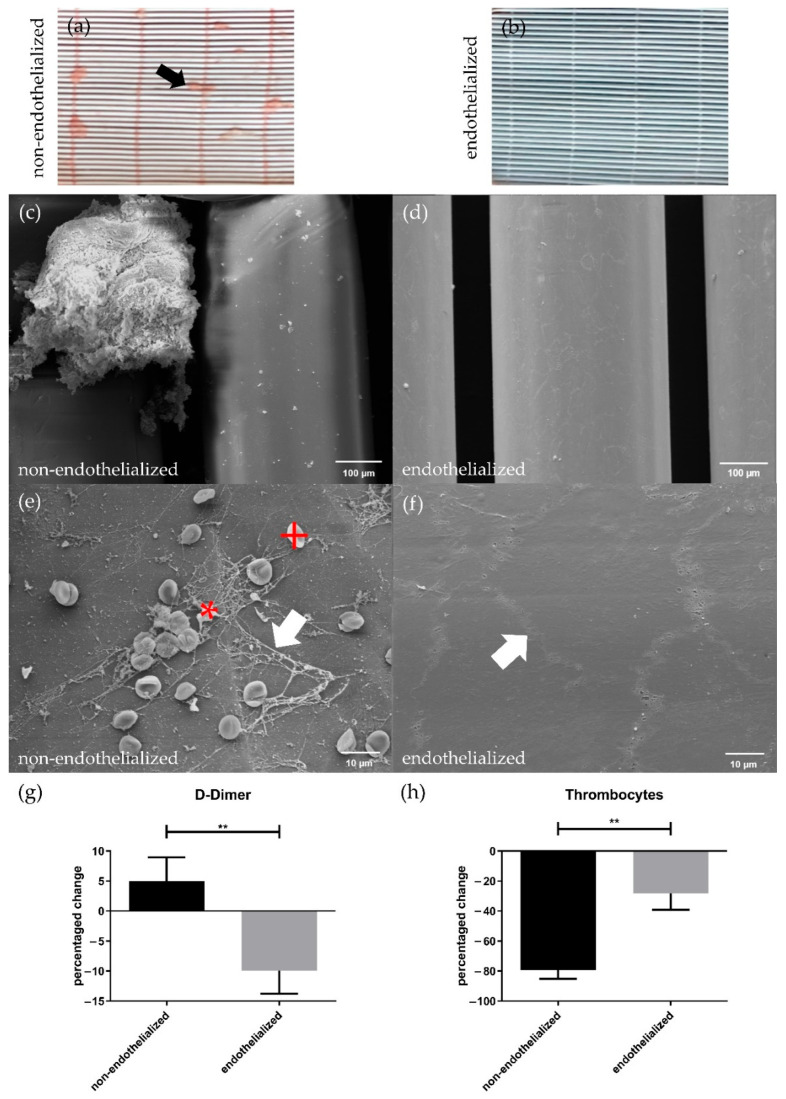
Thrombogenicity assessment of HFMs with and without pCEC monolayer after blood flow exposure. Macroscopic examination of (**a**) non-endothelialized and (**b**) endothelialized HFMs; (**a**) black arrow = macroscopically visible thrombus; SEM images of (**c**,**e**) non-endothelialized and (**d**,**f**) endothelialized HFMs; (**e**) white arrow: fibrin, red +: erythrocyte, red *: thrombocyte; (**f**) white arrow: cell-cell contact within confluent EC-monolayer; (**g**) D-Dimer and (**h**) thrombocyte count level changes in the non-endothelialized and endothelialized HFM group before and after blood flow exposure. An unpaired *t*-test was used to compare both groups. Results are given in mean difference [%] with SD (*n* = 3) (** *p* < 0.01).

The results after blood flow exposure revealed striking differences between endothelialized and non-endothelialized HFMs. While the non-endothelialized HFMs showed the formation of several macroscopically visible thrombi after blood perfusion (Figure 6a), endothelialized HFMs lacked any thrombus deposition at all (Figure 6b). These findings were confirmed by SEM, where additionally also no formations of microscopic thrombi on the endothelialized HFMs (Figure 6b,d) could be observed. Instead, SEM images indicated that fibrous network structures from coagulated fibrin with entrapped erythrocytes and platelets occurred only on non-endothelialized HFMs (Figure 6c,e). Moreover, SEM also confirmed the integrity of the pCEC monolayer, including the presence of intact cell-cell connections between the endothelial cells after blood flow exposure (Figure 6f).

As an additional indicator for induced coagulation processes, D-Dimer levels were measured before and after blood flow exposure of endothelialized and non-endothelialized HFMs. Here, the concentration of D-Dimers in the blood from circuits containing the non-endothelialized HFMs increased during flow exposure by 9.926%. Conversely, and significantly different to the non-endothelialized HFMs (*p* < 0.01), a drop in the D-Dimer concentration by −5.020% (Figure 6g) was measured for endothelialized HFMs.

A reduced thrombogenicity of endothelialized HFMs was also confirmed by determination of the thrombocyte count in blood before and after flow application. The number of non-adhered thrombocytes in the blood decreased drastically by −79.36% after contact to non-endothelialized HFMs, while the loss by adhering thrombocytes was significantly less prominent at −28.20% after blood exposure to endothelialized HFMs (*p* < 0.01) (Figure 6h).

## 4. Discussion

With the view to develop the BHL as an alternative therapy option to LTx, several studies have been published in recent years, proving the general feasibility of this intention. Representing a core achievement in BHL development, our group was able to establish a confluent endothelial monolayer (EML) on PMP HFMs, used in contemporary ECMO oxygenators [14]. Following this, we were able to identify and establish suitable surface coatings [16,17] and refined seeding protocols [15]. Additionally, we focused on gas transfer efficiency analysis indicating no impact of endothelial cells on the membrane surface [20,24]. Moreover, different studies demonstrated that the neo-endothelium, established on the HFM, remains viable, anti-thrombogenic, non-inflammatory and resilient under clinically relevant flow conditions that were simulated with endothelial cell culture medium even in long-term evaluation [14,17]. Furthermore, we identified two potential candidates for EC sources that could be safely applied for future clinical use of the BHL without the risk of acute rejection, i.e., ECs derived from induced pluripotent stem cells [21] and MHC-Class-I silenced human ECs [22].

With these recent promising results, the next important stage along BHL translation is the in vivo assessment. This is essential to analyze the envisaged performance of the device under clinical conditions, inter alia for crucial bio- and hemocompatibility analysis. Indeed, short-time exposure of endothelialized gas exchange membranes with whole blood has already been performed, but in a xenogeneic setting, which only provides very limited information for the clinical application of HFMs [19]. However, for experiments focusing on immunological reactions towards the prospective source of ECs, such as MHC-silenced ECs [22] or iPS-ECs [21], and also in parallel to specific lung injury models to mimic and investigate gas exchange capacities in patients with end-lung diseases, BHL testing in an allogeneic animal model, especially for long-term performance, is mandatory.

For this purpose, the porcine model is well-suited, as pigs have comparable anatomical and physiological properties to humans. In general, organ sizes and relative organ perfusion rates [25], but also the immunological system [26], are comparable, which are important prerequisites for future translation. Additionally, blood coagulation is very similar [27,28], with the exception that porcine blood is hypercoagulable compared to humans [29,30]. Indeed, this needs to be considered with care when assessing anti-thrombogenic effects of the EML in future in vivo experiments. However, this represents only a minor limitation of the pig as animal model, since a confirmation of the positive anti-thrombogenic and anti-coagulative effect of the porcine EML, in the demanding porcine setting, may indicate that this desired effect might be even more pronounced in the human scenario. However, the pig seems to be the best fitting animal model as multiple porcine lung injury models have also been reported and can be used for BHL assessment [31,32,33].

Analyzing the BHL in the porcine model entails that the biological components, i.e., ECs, need to come from porcine sources and not as in all our preceding studies from human sources, to prevent potential xenogeneic reactions, which would distort the results around BHL application [34,35]. Furthermore, analysis tools, e.g., antibodies or primer pairs, need to be adjusted to detect the porcine biology. Therefore, the goal of this study was to prepare for this translational step, i.e., testing the BHL in vivo, by establishing and assessing our endothelialization approach for the HFM, using porcine ECs.

Therefore, we established our easy to handle and reliable EC isolation and cultivation protocol from porcine carotid arteries, enabling the generation of a viable and confluent monolayer of high endothelial purity on the HFM. On the one hand, it is important to have a pure neo-endothelium as the anticipated anti-thrombogenic property will be most effective, when endothelial cell surface molecules, which actively control hemostasis, are abundant [36,37]. On the other hand, it is crucial, as complete cellular coverage of all artificial surfaces will prevent the adsorption of coagulation-initiating proteins. Additionally, the gapless integrity of the porcine EML was evidenced by a strong and ubiquitous fluorescence signal for the detection of the intercellular junction protein VE-Cadherin, which was also detectable after several subcultivations. As the EC population could also be subcultivated repeatedly without massive loss to viability, phenotype or doubling time, the efficiency of our protocol to generate the needed large number of ECs (more than 5 × 10^8^) to cover the prospective porcine BHL prototype, was proven. Furthermore, ECs were still able to synthesize the basal lamina protein fibronectin de novo, which is essential in signaling a native-like environment, mediating a physiological EC behavior and contributes to the EML stability under flow conditions, as observed recently with human ECs on fibronectin-coated HFMs [17].

However, being a physiologically crucial contributor in hemostasis regulation, ECs in the native blood vessels can also enter an activated, pro-thrombogenic state, to initiate blood clot formation, e.g., needed for wound closure at EML injury. In addition, pro-inflammatory cytokines can induce the endothelial pro-inflammatory state [38], which is an essential physiological response for the leukocyte recruitment towards inflamed tissue to trigger rejection processes [39]. Moreover, excessive inflammatory responses can also result in EC apoptosis and EC detachment [40]. Thus, both pro-thrombogenic and pro-inflammatory processes should not be triggered accidentally by the EC isolation nor by contact of the ECs to the foreign membrane surface or any other condition during BHL application, for the tissue engineered monolayer to prevent thrombotic occlusion, systemic inflammation or EC detachment. In order to rule out an injury or stimuli mediated shift of the porcine ECs towards these undesirable EC states, we measured the gene expression levels of relevant genes. Under standard culture conditions, porcine ECs resided in a non-activated state but were still able to respond physiologically to deliberate TNFα stimulation by upregulation of the pro-inflammatory genes E-Selectin, ICAM and TF and the downregulation of TM. The behavior of the porcine ECs and their response to TNFα was very similar to what we observed in our earlier studies with anti-thrombogenic human ECs [14,17], indicating that the insights on the reaction of the porcine EML during future BHL application can also predict the behavior of the EML in the prospective human BHL.

After proving the general applicability of the porcine ECs, we moved on to endothelialize H/A-coated HFMs, applying our seeding protocol established for human ECs [17]. Hereby, monolayer establishment was as effective as with human ECs [14,15,16], without any necessary adjustment to cell number or cultivation procedure. A viable and confluent porcine EML was detectable all over the HFM, and neither seeding procedure nor HFM surface substrate change, i.e., H/A, mediated a shift towards cellular activation. In accordance with physiological behavior and as detected on TCP, significant upregulation of E-Selectin, ICAM and TF was only noted upon deliberate TNFα activation. When seeded on HFMs, expression levels of TM were slightly but significantly higher compared to TCP, indicating a minute influence probably caused by the flow evoked by the rotation during the seeding procedure [41]. However, the higher TM expression at this stage would have no major implications. On both, TCP and HFM, ECs synthesized the extracellular matrix protein fibronectin de novo, suggesting that the ECs are potentially able to recreate their native basal lamina niche, covering over time the surface they initially adhered to, and thereby also returning to a native-like phenotype. Furthermore, the synthesized basal lamina like matrix secreted on the artificial surface may also be beneficial to provide physiologic cues needed for the efficient re-population and EC migration towards HFM areas, where ECs were accidentally worn off. Here, the pCECs and the human ECs, assessed earlier, also shared the ability to synthesize basal membrane proteins, although for the latter, collagen-IV, a different important basal membrane protein, and not the fibronectin deposition was confirmed [17].

Next, we assessed the general propensity of the porcine EML on the HFM to withstand clinically relevant flow conditions, simulated with culture medium. In order to keep the comparability to earlier studies performed with human ECs, we applied flow rates of 15 mL/min, which represented the scale-adjusted flow rate during a clinical ECMO setting at 30% cardiac output (1.5 L/min) [14,42]. Similar to human ECs, no appreciable cell detachment was detected [17]. Additionally, comparative gene expression analysis revealed on the one hand physiological responsiveness to flow conditions by KLF2 upregulation [43,44]. On the other hand, E-Selectin and ICAM levels were not influenced by fluid flow, whereas TF expression was elevated, but did not indicate a switch towards the pro-coagulative state and rather represents a higher baseline expression under these conditions, as seen already in human ECs [17]. Additionally, TM levels were slightly downregulated in porcine ECs upon flow exposure, which, however, did not fall below the level of deliberately activated porcine ECs on TCP, and thus, could be neglected at this point.

Gene expression analysis obtained the first piece of evidence that pCECs resided in the endothelial non-inflammatory and non-thrombogenic state that is beneficial to actively inhibit thrombus formation on HFMs. However, as cellular functionality is decisive for this attribute, complementary hemocompatibility assays were performed, using heparinized porcine blood for the first time. Prerequisite for this analysis was the proven EML flow resistance under clinically relevant conditions. Based on former promising results regarding flow resilience using 15 mL/min, achieved by both human and porcine ECs, flow rates were increased to 60 mL/min representing about 100% cardiac output [45]. Moreover, exposure time was limited to six hours to keep the potential influence of in vitro factors, such as nutrient consumption, hemolysis or pH shifts, within acceptable limits [46]. Despite increased shear stress, induced by the higher viscosity of whole blood [47], the EML also withstood clinically relevant flow exposure, suggesting its resistance during in vivo BHL application. These promising results were not anticipated, as earlier studies with human ECs indicated a noticeable cell detachment at higher flow rates, when seeded on H/A HFMs instead of fibronectin pre-coated HFMs [14,17]. Thus, and due to the fact that in this study fibronectin deposition on the HFM surface by pCECs could be detected, we hypothesize that pCECs may produce fibronectin, or extracellular matrix in general, more efficiently or faster than human ECs, which manifested in a more stable and resistant monolayer. The difference in extracellular matrix synthesis kinetics between human and porcine ECs and the implications for cell adhesion strength under flow conditions will be the subject of future experiments.

Then, the proceeding functionality assay indicated the anti-thrombogenic EML state, already appreciable by eye, but also impressively confirmed by SEM images by the absence of fibrin deposits and thrombocytes on endothelialized HFMs, proving the improved HFM hemocompatibility, which is crucial for reliable BHL long-term application. In contrast, and comparable to the situation frequently seen during clinical application of ECMO with human patients, multiple thrombus-like aggregates attached to the non-endothelialized HFMs, even though the blood in both settings was fully heparinized [48]. Additionally, the anti-thrombogenic effect of the porcine EML was detectable by changes in D-Dimer levels, cleaving products of already formed fibrin clots, which significantly increased in blood exposed to non-endothelialized HFM, while application to endothelialized HFMs resulted in a significant decrease, indicating the inhibition of fibrin clots by the monolayer. Likewise, the anti-thrombogenic effect was confirmed by the respective thrombocyte counts, as a significantly lower decline was observed for the endothelialized HFMs, indicating the prevention of thrombocyte binding. Nevertheless, the experimental set-up consisted of many blood-contacting components, such as tubing and connectors, and utilized a peristaltic pump, which together may have been responsible for the general thrombocyte decrease in both approaches. Based on the aforementioned potential hypercoagulability of porcine blood, the coagulation and thrombocyte adhesion evoked by the non-endothelialized surfaces in this study may be less pronounced when repeated with human blood. Although the efficient inhibitory effect of the human EML on platelet adhesion to the HFM could be proven in earlier studies [16,18,21], a direct comparison between porcine and human blood in this regard will be subject of future studies. However, the witnessed propensity of the non-endothelialized surfaces within the mock circuit to potentially attract thrombocytes and trigger the coagulation system underscores the clear need not only to improve HFM, but also complete BHL component hemocompatibility for safe long-term application as an alternative to LTx.

Finally, the observed in vitro similarity of porcine and human EC behavior is essential for the forecasting power and reliability to translate gained data of the future porcine in vivo model to human prototype development and application. As we considered the implementation of all mentioned assays mandatory before putting any animals at risk by performing ill-prepared in vivo experiments [49], we will now move forward with a clearer conscience towards porcine BHL application, based on the very good results achieved. Future animal studies will inter alia include the application of allogeneic, genetically modified porcine ECs, following the already established MHC-silencing of human ECs, which potentially will be used as clinically relevant and universal EC source, as they proved their capability to escape a potential host immune-rejection in vitro [22].

In summary, we managed to establish a first porcine in vitro model of the BHL. We succeeded in establishing an EML from pCECs on the HFMs of the prospective BHL. The viable and fully confluent EML showed excellent resilience towards clinically relevant flow conditions, even when flow was generated by whole blood. Moreover, EML could significantly improve the hemocompatibility of HFMs as witnessed by the elimination of blood clot formation and reduction of thrombocyte adhesion, which is urgently needed for enabling long-term application of BHL as a bridge and alternative to LTx and may render partly lethal anticoagulative therapy unnecessary. Thus, the positive outcome of this preparation study now justifies taking further steps towards the pre-clinical translation of the BHL, i.e., testing in the porcine model under healthy but also lung injury models, reflecting the clinical scenario in end-stage lung diseased patients.

## Figures and Tables

**Table 1:** Antibodies used for immunofluorescence staining.

Antibody Name	Dilution	Vendor
Anti-VE Cadherin (ab33168)	1:300	Abcam, Cambridge, UK
Anti-Fibronectin (ab45688)	1:250	Abcam, Cambridge, UK
Rabbit IgG Isotype Control (ab172730)		Abcam, Cambridge, UK
Donkey anti-rabbit Cy2	1:100	Jackson ImmunoResearch, Ely, UK

**Table 2:** Primer pairs used for gene expression analysis.

Gene Name and ID	Primer 1	Primer 2
ß-Actin XM 003124280.4	GATCAAGATCATCGCG- CCTCC	GGAATGCAACTAACAG- TCCGCC
Endothelium-Selectin (E-Selectin)NM 214268.2	TCCTGTCAACGGAGTC- GTGA	GTCACAGCTTTACACGT- TGGC
ICAM-1 (ICAM) NM 213816.1	GCTCAGTGTCCTGTAT- GGACC	AGAGCTGGTGGCCTGA-CATT
Thrombomodulin (TM) NM 001130732.1	CAACCAGACTTCGTG- CCCTG	GTAGCCGTTGTTGCAC- TCGT
Tissue Factor (TF) NM 213785.1	TTAGTCAGGGTGAAC- GGCAC	GGTCGTGGCCTTTTTC- TTTCC
von Willebrand Factor (vWF) NM 001246221.1	AGGGGGACCAAAGC- ATCTCC	TGAAAGTTGCCGCTC- CCATC
CD31 NM 01010101.01	CACGGAGGTCTGGAA- CAAAG	TCTGCTCTGCGGTCC- TAAGT
VE-Cadherin (VE-Cadh.) NM 001001649.2	GCGAGTTCACCTTGT- GCGAG	CGAGGAGGGAGATC- ACTGCG
Krüppel-like factor 2 (KLF 2) NM 001134351.2	CGTCTCCGCTGGAGC- TACTA	GTAGGGCTTCTCGCC- TGTAT
Collagen 4 subunit A1 (Col 4 A1) XM 021065910.1	ATGCAACGGGACAA- AGGGTG	CCCAGGTATGTGGCC- GAGTA

## Data Availability

Not applicable.

## Supplementary Materials

The following supporting information can be downloaded at: https://www.mdpi.com/article/10.3390/membranes12070687/s1, Figure S1: Schematic overview of the experiment, Figure S2: Characterization of the endothelial geno- and phenotype.
