# Supplementary material for: Towards Biohybrid Lung Development: Establishment of a Porcine In Vitro Model
Source: Membranes (Basel). 2022 Jul 3;12(7):687. doi: 10.3390/membranes12070687 (PMC9325277; doi:10.3390/membranes12070687)
Supplement: Supplementary file 1 [file membranes-12-00687-s001.zip › membranes-1730365-SM.pdf]

membranes

MDPI

Supplementary material for

Towards Biohybrid Lung Development: Establishment of a Porcine in vitro Model

Simon Schlör [1,2,+], Michael Pflaum [1,2,+], Klaus Höffler [1,2], Christian Kühn [1,2,3], Axel Haverich [1,2,3] and Bettina Wiegmann [1,2,3,4,*]

[1] Department for Cardiothoracic, Transplantation and Vascular Surgery, Hannover Medical School, Carl-Neuberg-Str. 1, 30625 Hannover, Germany; schloer.simon@mh-hannover.de (S.S.); pflaum.michael@mh-hannover.de (M.P.); hoeffler.klaus@mh-hannover.de (K.H.); kuehn.christian@mh-hannover.de (C.K.); haverich.axel@mh-hannover.de (A.H.)
[2] Lower Saxony Center for Biomedical Engineering, Implant Research and Development (NIFE), Stadtfelddamm 34, 30625 Hannover, Germany
[3] German Center for Lung Research (DZL), Carl-Neuberg-Str. 1, 30625 Hannover, Germany
[4] Member of the DFG priority program SPP 2014
+ These authors contributed equally to this work
* Correspondence: wiegmann.bettina@mh-hannover.de

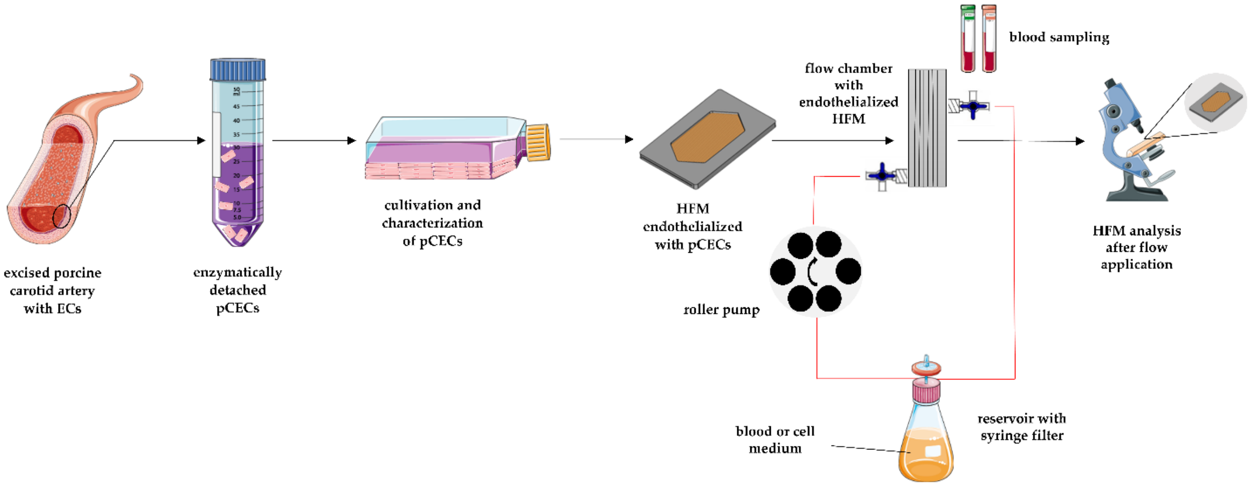

Supplement figure S1. Schematic overview of the experiment. pCECs: porcine carotid artery endothelial cells; HFM: Hollow Fiber Membrane.

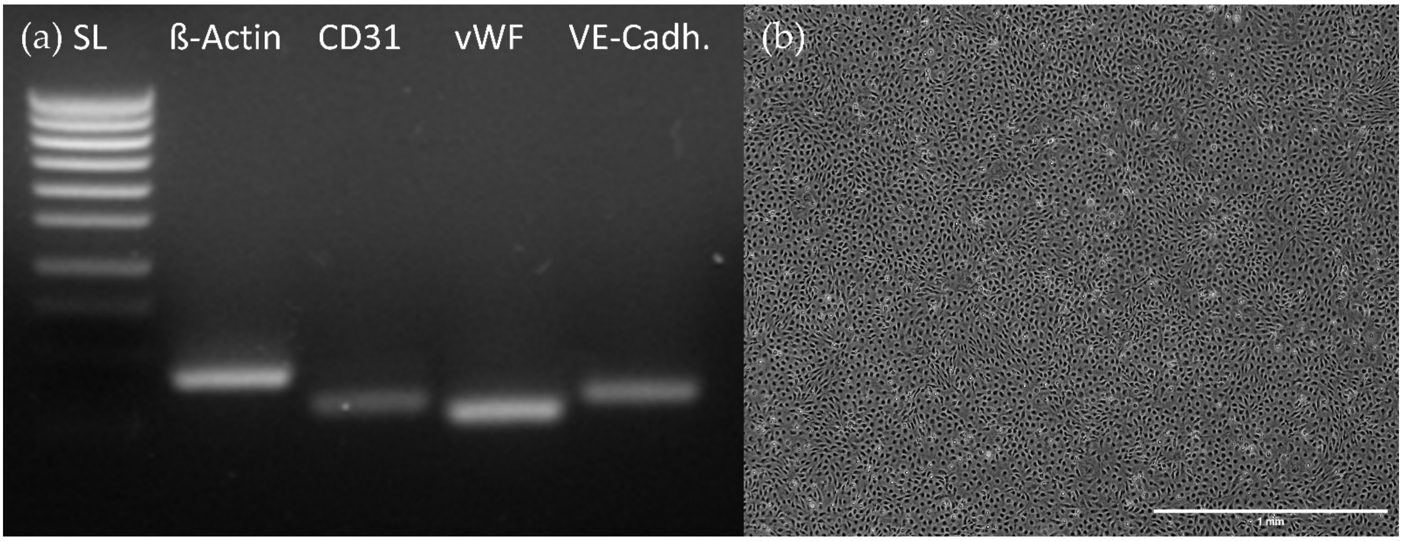

Supplement figure S2. Characterization of the endothelial geno- and phenotype**.** (**a**) RT-PCR for the qualitative expression analysis of the endothelial specific CD31, vWF and VE-Cadherin. SL: standard ladder, vWF: von Willebrand factor, VE-Cadh.: vascular endothelium-cadherin; (**b**) phase contrast microscopy showing the typical cobblestone morphology of the confluent EC-monolayer.
